# Hospitals accreditation status in Indonesia: associated with hospital characteristics, market competition intensity, and hospital performance?

**DOI:** 10.1186/s12913-019-4187-x

**Published:** 2019-06-11

**Authors:** Viera Wardhani, Jitse Pieter van Dijk, Adi Utarini

**Affiliations:** 1 Post Graduate Program in Hospital Management, Faculty of Medicine, Universitas Brawijaya, East Java, Jalan Veteran No 1, Malang, 65145 Indonesia; 2 Doctoral Program in Medicine and Health, Faculty of Medicine, Public Health and Nursing, Universitas Gadjah Mada, Jalan Farmako Sekip Utara, Yogyakarta, 55281 Indonesia; 3 Department of Community and Occupational Medicine, University Medisch Centrum, University of Groningen, Antonius Deusinglaan 1, 9713 AV Groningen, The Netherlands; 4 Department of Social Medicine and Public Health, Faculty of Medicine and Dentistry, Palacký University, Hněvotínská 3, 775 15 Olomouc, Czech Republic; 5 Department of Health Policy and Management, Faculty of Medicine, Public health and Nursing, Universitas Gadjah Mada, Jalan Farmako Sekip Utara, Yogyakarta, 55281 Indonesia

**Keywords:** Hospital accreditation, External evaluation, Organisational design factors, Market competition, Hospital performance, Indonesia, Developing countries, Organizational factor, Market intensity, Determinant

## Abstract

**Background:**

Hospital accreditation is widely adopted as a visible measure of an organisation’s quality and safety management standards compliance. There is still inconsistent evidence regarding the influence of hospital accreditation on hospital performance, with limited studies in developing countries. This study aims to explore the association of hospital characteristics and market competition with hospital accreditation status and to investigate whether accreditation status differentiate hospital performance.

**Methods:**

East Java Province, with a total 346 hospitals was selected for this study. Hospital characteristics (size, specialty, ownership) and performance indicator (bed occupancy rate, turnover interval, average length of stay, gross mortality rate, and net mortality rate) were retrieved from national hospital database while hospital accreditation status were recorded based on hospital accreditation report. Market density, Herfindahl-Hirschman index (HHI), and hospitals relative size as competition indicators were calculated based on the provincial statistical report data. Logistic regression, Mann-Whitney U-test, and one sample t-test were used to analyse the data.

**Results:**

A total of 217 (62.7%) hospitals were accredited. Hospital size and ownership were significantly associated with of accreditation status. When compared to government-owned, hospital managed by ministry of defense (B = 1.705, *p* = 0.012) has higher probability to be accredited. Though not statistically significant, accredited hospitals had higher utility and efficiency indicators, as well as higher mortality.

**Conclusions:**

Hospital with higher size and managed by government have higher probability to be accredited independent to its specialty and the intensity of market competition. Higher utility and mortality in accredited hospitals needs further investigation.

**Electronic supplementary material:**

The online version of this article (10.1186/s12913-019-4187-x) contains supplementary material, which is available to authorized users.

## Background

Hospital accreditation programmes are avenues through which a complex policy intervention functions to promote adherence to quality and safety management standards and drive continuous quality improvement. On a more practical level, these programmes represent a quality management system (QMS), total quality management (TQM) or continuous quality improvement (CQI) standards that should lead to an improvement of the hospital’s overall performance [1–3]. While accreditation has been widely adopted in healthcare organizations, the history of quality management theory and accreditation was initiated in manufacture industries with different organizational culture and environment [4, 5], that are the two important determinants for the adoption of QMS implementation [6, 7]. Knowledge gaps, therefore, remain existing and raise questions about whether the adopted theory, standards, and practices outside the health care industries will fit in and whether it has an impact on the overall health care organization performance as well as on patient satisfaction [8–10].

A quality management system is the key important link between hospital accreditation and the end point of quality and safety. Hence, previous studies have identified certain organisational factors needed for successful adoption of QMS standards such as strong leadership, continuous quality improvement, and human resource development [6, 7]. Studies of these factors have also identified that internal organisational factors (size, ownership, culture, leadership, and technical capability) and of environmental factors (health system and market competition intensity) play as driving forces for QMS implementation as well as hospital accreditation [6, 11, 12].

Aside from hospital characteristics, healthcare market competition is one of the external factors that drive hospital quality. Previous studies found that in a highly competitive market, hospitals face more pressure for quality improvement efforts [13, 14]. In such an environment, the hospital accreditation status adds a competitive advantage since it is viewed as a hospital quality indicator that is considered by patients, referral doctors, and other purchasers [15–17]. In Indonesia, hospital accreditation status is required as a credential indicator by the national health insurance agency [18]. Furthermore, the sustainability of healthcare accreditation highly depends on government support, market size, funding, and continuous evaluation of the accreditation programme and standards [6, 19–21].

The implementation of hospital accreditation in Indonesia as a mechanism of external quality assurance was initiated and has been ongoing since 1995. The programme is managed by the Indonesia Commission on Accreditation of Hospitals (ICAH) as a formal government agency for hospital accreditation, which later became a more independent agency. The earlier Indonesian hospital accreditation standard entailed of three different schemes, based on the number of service unit/department evaluated during the survey, i.e. basic (5 service unit), advance (12 service unit), and full accreditation (16 service unit) [22]. Started in 2013, the Joint Commission International (JCI) hospital accreditation standard that focused more on the process of care with patient safety as the ultimate goal and considered hospitals as an integrated system was adopted. Thus hospitals could not be evaluated as separated services [18, 22].

Intended as a safeguarding mechanism, policies which were started after mandatory policy for hospital accreditation under the Indonesian Hospital Act (2009) have intensified the external pressure for hospitals [23]. The urge to implement the international standards adopted from (JCI) which began in 2013 added more pressure for hospitals to apply for accreditation [24]. Moreover, considering that removing physical and financial barriers to health care facilities will not guarantee the outcome when it is provided in substandard care [25, 26], accreditation also required as a credentialing mechanism for healthcare providers under universal health care quality coverage policy [22, 24].

As the basic intention of accreditation is to improve hospital quality performance, several studies have been carried out to evaluate its benefit. Systematic reviews of the health sector accreditation’s impact have identified two areas that consistently benefit from the accreditation: promoted organisational change and professional development [8, 10, 27, 28]. These studies have found that the association between accreditation and organisational performance, financial indicator, quality measures, and programme assessment was inconsistent, and the correlation with patient satisfaction was not sufficiently conclusive [9, 10, 16, 27, 28]. Hinchcliff et al. concluded that based on a limited amount of evidence, a potential correlation existed between accreditation and high-quality organisational process as well as clinical care [28].

Changes in hospital performance can be approached based on the quality dimension. The World Health Organisation (WHO) and the Institute of Medicine (IOM) suggested the following quality dimensions to describe hospital performance: accessibility, efficiency, effectivity, acceptability (patient-centred), equity, and safety [29]. At the hospital level, bed occupancy rate (BOR), turn over interval (TOI) and average length of stay (ALOS) are widely used to describe the hospital capacity, its utilisation efficiency. These indicators also indirectly represent accessibility [23, 30], and, at the health system level, are also used to measure the system’s capacity to serve and provide access to care. In addition, the gross mortality rate (GMR) and net mortality rate (NMR) are widely reported as the hospital-based indicators that describe overall patient outcome and clinical effectiveness [29, 31].

Studies of the effect of hospital accreditation are still characterised by a lack of strong and consistent evidence regarding the benefit of accreditation on clinical performance in particular [8, 10, 28, 32]. In addition, most of those studies, which have been performed in developed countries, are calling for studies in developing countries. A qualitative report of hospital accreditation programmes in low and middle-income countries has identified a need to describe hospital accreditation standard elements both in terms of successful implementation and its relation with hospital performance [19, 20, 33].

As the fourth most populous country in the world, Indonesia faces a serious population maldistribution resulted in wide socio-economic disparities. Almost 70% percent of its inhabitants are condensed in one island, Java, which is only equal to 11,5% of Indonesia land area, while the rest are sparsely distributed in others 17.000 islands [34]. Under decentralized health system policy, each province and district have financial and operational authority. Most of the responsibilities regarding health care were transferred to the district level while the provincial government is only responsible for the coordination of referral care between districts [35]. While decentralization is intended to reduce disparities and develop local capabilities, the latest studies found that the disparities remain exist make it not easy to be treated equally [36, 37]. Hence, in a country with extreme diversity such as Indonesia, studying health system at the national level needs to be very carefully performed [38, 39]. Bearing in mind these circumstances, our study is intended as a starting point for further research to have a better understanding of health system at the national level. For that reason, we selected East Java, a province with 38 districts and 39.3 million inhabitants (16% of Indonesian) that has more similar characteristics with other provinces outside Java [34, 40]. Furthermore, compared to other provinces, East Java has fairly balance development in term of income distribution and regional equity [40].

This study explores the association between organisational design factors and market competition intensity with the hospital accreditation status. We further examine the difference in hospital performance indicators across their accreditation status. The findings from this study will provide supporting evidence towards understanding the link between organisational design, accreditation status and hospital performance in developing countries.

## Methods

### Sample

In this study, we selected one province, East-Java, as one of the provinces in Indonesia that has a middle socioeconomic status as the dominant province characteristic [34]. A total of 346 hospitals in East-Java listed in the National database by the year 2014 were included in this study. These hospitals represent 17% of the national hospitals, and 56% are publicly owned hospitals distributed in 38 districts.

### Measures

The organisational design factors and latest accreditation status data were retrieved from online national hospitals database managed by Directorate General of Medical Service Ministry of Health [41] and hospital accreditation report provided by ICAH [42]. Three measures were used to describe organisational design factors, i.e., hospital size, ownership, and specialty of service provided. Hospital size was measured as four ordinal scales referring to the number of hospital beds classification, i.e., 1) ≤50; 2) 51–100; 3) 101–200; 4) > 200. The hospital ownership was differentiated based on the organizational culture and public or private nature characteristics of its owner, starting from more public-oriented with strong bureaucracy and hierarchy culture to more profit-oriented hospitals, as follows 1) public hospitals, 2) military-managed, 3) state-owned enterprise managed, and private owned hospitals. Thirdly, the specialty of services provided was defined as the hospital specialty type 1) general hospitals, 2) maternal and child-care hospitals, and 3) other ranges of specialist hospitals (i.e., surgery, orthopaedic, dentistry, and psychiatric care). In addition to organisational design factors, the number of specialist physicians was used as the measure for service capacity. A dichotomous scale, accredited and not-accredited, was used to differentiate hospitals accreditation status.

The market competition intensity was calculated based on the 2014 East-Java Provincial Bureau of Statistic report. Competition was approached based on two dimensions: *market density* that represents the number of hospitals in a determined market area, and *market concentration* that focuses on the distribution of the market share of a comparable product. The market position that differentiates a hospital’s characteristics from the others is a significant measure for market competition for rural hospitals. Therefore, we used those three measures to describe the *hospital market competition intensity,* namely: 1) the number of hospitals with the same specialty type in one district as a measure of market density, 2) the Herfindahl-Hirschman index (HHI) as a measure of market concentration, and 3) the relative size of a hospital as a measure of market position. The HHI is calculated by summing the squared market shares for all hospitals in the same market, defined as the hospitals located in the same district. The market share is defined as the proportion of the number of hospital beds in each particular hospital in comparison to the total hospital beds in the market [11, 43]. The theoretical value of the HHI can range from close to zero to 10,000 or 100%. When the HHI value is above 1800, the market is said to be highly concentrated. A hospital’s relative size was measured as a hospital’s number of beds relative to the average number of hospital beds within the same type and district. The district in terms of both administrative and geographic boundaries was selected as the defined market area that referred to the health insurance referral area. Hence, competition was defined for hospitals with the same type of service within a district [43, 44].

Hospital performance data were obtained from the national hospital registry managed by the Ministry of Health. Every hospital is responsible for entering and updating the performance data regularly. However, hospitals do not generally comply with this requirement, which affects the completeness of the data. Of the 346 hospitals in East Java province, less than 50% (22–47%) submitted their reports on hospital performance indicators. Five available indicators were retrieved, namely utility and efficiency indicators, i.e., 1) *bed occupancy rate* (BOR), 2) *average length of stay* (ALOS), 3) *turnover interval* (TOI) and clinical indicators, i.e., 1) *gross mortality rate* (GMR) and 2) *net mortality rate* (NMR). The comparative hospital performance standards were set based on the 2008 Hospital Minimum Service Requirement.

### Statistical analyses

A table stratified by hospital accreditation status and its related factors is provided to describe the hospital characteristics across accreditation status. Furthermore, logistic regression was performed to analyse the role played by organisational design factors and market competition intensity in explaining hospital accreditation status. For the logistic regression analysis, of the total 346 hospitals, 4 cases with very high values (number of specialist physicians > 200 and the number of hospital beds > 700) were excluded. Finally, we compared the clinical performance indicators on 1) between accredited and not accredited hospitals and 2) between both accredited and not-accredited hospitals with the national threshold in each indicator. Since the data were not normally distributed, we used the Mann Whitney U-test for the first purpose, while for the second purpose we used one sign test with the median of each comparable group. Considering that the national threshold for clinical indicators was provided in a range value, the comparable values for the one sign test were the mid value of each threshold as follows: 1) BOR = 75%; 2) ALOS = 7,5; 3) TOI = 2,5; 4) NMR = 2; 5) GMR = 4,5.

We performed multivariate multiple imputation (SPSS v 24), to impute missing values. Of the total 12 studied variables, 7 variables have varied amount missing values from 7,2% (number of specialist physician) to 77,2% (net mortality rate). All variables that were included in the analysis (logistic regression and comparative analysis) were included in the predictive model. Predictive mean matching was used to impute scale variable and considering that the highest missing values is 77.2% we created 80 imputed data set, with maximum iteration was set at 80. The distribution of the variables with missing data did not differ substantially between completed and multiple imputation data set (Additional file 1: Table S1). Result of the logistic regression analysis and comparative analysis obtained from analysis with complete data (Additional file 2: Table S2) also did not substantially differ with the same analysis obtained from multivariable multiple imputed data set.

## Results

Hospital size and ownership and the number of specialist physicians were significantly associated with a higher likelihood of a hospital being accredited. The hospital specialty types, the number of specialist and market density did not associate with the accreditation status. Although not statistically significant, accredited hospitals tended to have a higher BOR and ALOS, though they also had a higher mortality ratio.

### The role of organisational design factors and market intensity in determining hospital accreditation status

Table 1 shows that of the different organisational design factors, size, and type of ownership were significantly associated with different accreditation status, while the number of specialist and market concentration were not. Compared with non-accredited hospitals, accredited hospitals were bigger and were generally government-owned. Accredited hospitals also had a higher human resources capacity as shown by a higher number of specialist physicians and nurses compared with non-accredited hospitals. Overall, all hospitals were in a high market concentration or low competition category irrespective of their accreditation status, even though the number of hospitals in the areas with accredited hospitals was higher (Table 1).

**Table 1:** Hospital accreditation status related to studied factors, in total and by hospital accreditation status

Studied Factors	Total	Accredited	Not Accredited	*p* value
		N (%)	N (%)	
Organizational design				
a. Size (number of beds)				< 0.001
□ ≤ 50	108	39 (36)	69 (64)	
□ 51–100	117	74 (63)	43 (37)	
□ 101–200	67	54 (81)	13 (19)	
□ > 200	54	50 (93)	4 (7)	
b. Ownership				
□ Public	69	52 (75)	17 (25)	< 0.001
□ Military	30	24 (80)	6 (20)	
□ State owned	12	11 (92)	1 (8)	
□ Private	235	130 (55)	105 (45)	
c, Specialty				< 0.001
□ General	253	178 (70)	75 (30)	
□ Maternity	67	27 (40)	40 (60)	
□ Specialist	26	12 (46)	14 (54)	
d. Number of specialists: mean (SD)	19 (27)	23 (33)	11 (11)	0.001
Market Competition Intensity: mean (SD)				
a. Market density	11 (10)	11.9 (0.77)	9.9 (0.89)	0.147
b. Relative size	1 (0.8)	1.15 (0.06)	0.75 (0.03)	< 0.001
c. HHI (%)	42.09%	64.5%	36.31%	0.244

Table 2 shows that hospital size and ownership status were significantly associated with the hospital’s accreditation status. The model is significant and explained 28.6% of the variance in the hospital accreditation status. Compared with small hospitals, hospitals with more than 100 beds had a higher probability of being accredited. Military and state-owned enterprise managed hospitals, but not private owned hospitals, when compared to public hospitals had a significantly higher probability of being accredited. (Table 2).

**Table 2:** Logistic regression analysis of the hospital accreditation status related to organizational design factors (number of beds, ownership status, specialty type, number of specialists), and market density

Explored Factors	B	SE	Wald	Df	Sig^a^	OR	95% CI for OR	
							Lower	Upper
Organizational design								
a. Hospital size								
● ≤ 50 (ref.)			9.155	3	0.027			
●51–100	0.875	0.335	6.837	1	0.009	2.399	1.245	4.622
●101–200	1.481	0.542	7.469	1	0.006	4.397	1.520	12.717
● > 200	2.136	0.978	4.774	1	0.029	8.466	1.246	57.511
b. Ownership status								
●Public (ref.)			8.254	3	0.041			
●Military	1.628	0.663	6.028	1	0.014	5.093	1.389	18.677
●State owned enterprise	2.103	1.157	3.302	1	0.069	8.187	0.848	79.067
●Private	0.531	0.454	1.368	1	0.242	1.701	0.698	4.144
c. Specialty service								
●General (ref.)			1.234	2	0.540			
●Maternity	−0.440	0.439	1.003	1	0.317	0.644	0.272	1.523
●Specialist	−0.393	0.500	0.619	1	0.431	0.675	0.253	1.798
a.Number of specialist physicians	0.013	0.014	0.805	1	0.370	1.013	0.985	1.042
Market Indicator								
a. Relative size	0.681	0.479	2.025	1	0.155	1.976	0.773	5.047
Constant	−1.460	0.604	5.843	1	0.016	0.232		

^a^*ref.* reference category

Hosmer and Lemeshow test *p* = .29; Nagelkerke R square = .286

### Comparison of the hospital performance indicators

Table 3 shows that there is no significant difference between accredited and non-accredited hospitals regarding utility, efficiency, and clinical indicators. Accredited hospitals tend to have a higher utilisation rate and concurrent mortality rate, though this did not reach significance. Although the mortality rate in accredited hospitals tends to be higher, when compared to the national standards requirements, the rate for all hospitals included in the comparison analysis regardless their accreditation status significantly exceeds the acceptable national standard.

**Table 3:** The difference of average hospital performance indicators BOR, ALOS, TOI, NMR, GMR from national standards and by accreditation status

Hospital Performance Indicator	1	2	3	p	p	p
	National Standard	Accredited Hospitals	Not-accredited Hospitals	(1–2)	(1–3)	(2–3)
	(mid value)	(SE)	(SE)			
BOR	75	52.62 (1.7)	44.43 (3.53)	< 0.001	< 0.001	0.133
ALOS	7.5	4.35 (0.34)	3.71 (0.25)	< 0.001	< 0.001	0,196
TOI	2	5.66 (0.93)	5.22 (1.52)	< 0.001	< 0.001	0.814
NMR	2.5	17.24 (1.30)	16.97 (2.55)	< 0.001	< 0.001	0.926
GMR	4.5	29.05 (2.14)	28.55 (3.57)	< 0.001	< 0.001	0.901

*BOR* bed occupancy rate, *ALOS* average length of stay, *TOI* turn over interval, *NMR* net mortality rate, *GMR* gross mortality rate

## Discussion

We investigated factors associated with hospital accreditation status, i.e., organizational design factors (size, ownership, specialty, and the number of specialist physicians) and market competition intensity as the drivers for hospital accreditation in East Java Indonesia. We also compared the differences in hospital performance indicators between the accreditation status.

Our findings show that hospital size and ownership are significantly associated with a higher likelihood of a hospital being accredited, while the hospital specialty types, the number of specialists, and market competition intention do not significantly relate to the accreditation status. Although not statistically significant, accredited hospitals tend to have a higher BOR and ALOS, though this is concurrent with a higher mortality ratio.

### The role of organisational design factors and market competition intensity in determining hospital accreditation status

We found that in our studied province, East Java, large, government-owned, and general hospitals have a higher probability of being accredited. Previous reviews have identified that, unlike small hospitals, large hospitals have more resource capacity; however, they also tend to have a hierarchical culture and more bureaucracy, which are a hindrance to the egalitarian culture needed to implement quality management and safety standards [28]. Organisational design factors such as size, ownership, and specialty describe the hospital’s capacity to serve the catchment area. They also simultaneously represent the organisation’s structure and culture and function as important determinants of the implementation of accreditation standards [6, 28]. The scarcity of resources, finances, and staff adequacy are defined as the capacity barriers faced by small hospitals, such as rural hospitals or hospitals in less developed countries, when implementing hospital accreditation standards [19, 20, 33]. Our findings emphasise the role that government support should play as the facilitating factor for overcoming the financial and resources barriers faced by hospitals while implementing the fundamental structure for continuous quality improvement [45, 46].

In terms of ownership, we also found that, compared with other government hospitals, military-managed hospitals have a higher probability of being accredited. The characteristic and structure of the owner is highly influenced by a hospital’s structure and organisational culture, which are important determinants of successful quality improvement strategies [6, 28]. While government hospitals tend to have more support and resources, they also exhibit a highly bureaucratic quality that hinders continuous improvement [6, 47]. The command structure in military-managed hospitals can be a hindrance to a culture of continuous quality improvement, but it can also act as a catalyst when innovation is diffused from the top down [6, 48]. Other studies related to ownership and hospital quality mostly divide ownership into ‘for profit’ and ‘not-for-profit’ and associate this trend with the organisation’s competitive behaviour [49]. The ownership characteristics of Indonesian hospitals are not strictly associated with for-profit behaviour. Mixed ownership characteristics might influence their relationship with quality improvement strategies and needs further investigation.

Furthermore, we found the number of specialist physicians was not related to the hospital’s accreditation status even though the number was slightly higher in accredited hospitals. Hospital accreditation standards require an adequate human resource capacity to be present for the structure or input standards of continuous quality improvement to be actualised. Most of the studies of hospital accreditation investigate the role or involvement of physicians, but not their actual number [27, 28, 50]. Those studies are performed in developed countries where the number of human resources is not a real problem, while in most developing countries the scarcity of human resources remains a huge problem [51]. Other studies in low and middle-income countries support the evidence that scarcity of finances and human resources remains the fundamental barrier at the structural level for having an effective hospital accreditation programme [45, 46].

We also found that market competition intensity has no significant associated with hospital accreditation status. A study of the impact of hospital competition on the inpatient quality indicators concluded that market competition had a positive unidirectional impact on the multidimensional nature of quality, especially in terms of visible aspects for the patient such as physician skill and expertise [17]. Other less visible indicators such as hospital structure and management, which mostly are measured as determinants for accreditation standard, were not found [17, 52]. In addition, the implementation of a universal health coverage in 2014 in Indonesia added additional pressure to pursue accreditation status, since accreditation was required by the health insurance provider as a measure for quality assurance. This finding supports previous evidence that a change in the accreditation policy has a significant impact on altering the balance and direction of hospitals’ market competition [11, 44, 53].

### The association between accreditation and hospital performance

Finally, we found no significant differences in hospital performance measures (utilisation or the mortality ratio) across accreditation status, though mortality rates were slightly higher in the accredited hospitals. These findings are in line with previous reviews showing inconsistent evidence between hospital accreditation, organisational performance, and patient outcomes [10, 27, 28, 54]. The DUQUE project concluded there was a consistent positive impact of accreditation on the process of care. However, though the benefit of accreditation on clinical outcome improvement is promising, the evidence is not consistent across studies [32]. The reason behind the higher mortality rate in accredited hospitals may be related to the fact that most referral hospitals are accredited. Compared with non-accredited hospitals, accredited hospitals are mostly large and government-owned and act as referral hospitals which have a greater share of severely ill patients. This pattern may lead to higher mortality and worse patient outcomes.

### Strengths and limitations

Many other studies evaluating the factors associated with hospital accreditation status have been conducted in developed countries with established health services and financial systems [8, 10, 27, 28, 54]. Within its limitation, this is the first study that evaluates the factors associated with hospitals accreditation status as well as its association with hospital performance indicators in one of the provinces in Indonesia, a developing country, and therefore adds evidence regarding the adoption and potential benefit of accreditation when resources are limited.

The limitation of using secondary data analysis such as problem with data completeness, though it could be managed using appropriate missing data analysis and imputation method, still call the need to improve hospital reported compliance by providing feedback and relating the report with positive consequences. Furthermore, using multiple imputation method will increase the opportunity of conducting a regular and continuous evaluation based on the available secondary data. In addition, the use of the net and gross mortality rates must be interpreted with considerable caution. There are many other factors beyond those that have been collected in this study that may explain differences or the lack of difference in these measures between accredited and unaccredited hospitals, such as the severity of the disease and patient age, which are strongly influenced by hospital classification and service type [31, 55]. Adjacent to that, the number of hospitals that had completed their performance data was considerably low that influence its representativeness. Hospitals with completed performance data mostly are accredited and have a higher size that could influence their performance.

Baseline data for accreditation status ultimately rely on interpretative data. However, data that form the basis of the accumulative score of hospital achievement in all accreditation standards do not consider the differences of specific successes and failures that occur during the fulfillment of each standard [55, 56]. Another problem arises from the limitations surrounding accreditation as a measure of the actual QMS implementation that will lead to quality improvement [57]. The accreditation evaluation was founded on short-term observation that was mainly based on the documented evidence. Since there is no cohort evaluation before and after the application for accreditation, the possibility exists that the accredited hospitals may discontinue the implementation after accreditation [56].

Strictly speaking, because we surveyed one province of Indonesia, our findings are not necessarily valid for other provinces. However, since East Java is one of the provinces in Java-Bali islands, the main capital area, that has a more comparable socio-economic profile with the other provinces outside Java-Bali [36], our finding may be potentially useful to other Indonesian provinces. As health care systems are nationally organised, it is difficult to extend similar observations to other low and middle income cuntries (LMICs) in South East Asia. Still, our results may tentatively provide some insights into other LMICs in South East Asia that mostly undergo a health system transformation through a decentralisation strategy and use hospital accreditation as a quality regulation tool [26, 58].

### Recommendation

Our finding that being a large, government-owned hospital is significantly associated with a higher likelihood of being accredited indicates a stronger role for government as a driver for accreditation. However, with the increasing role of private health care, a government takeover of ownership is not a realistic option. The government should mainly oversee either the process of quality assurance or the reporting mechanisms for both public and private health care. Hospital benchmarking should be based on standardised performance reports that are linked to a hospital’s credentialing mechanism [59, 60]. Such a system will strengthen the accountability of health care.

Small hospitals must get support for basic requirements such as human resources [45, 46]. A new regulation is necessary to better distribute human resources with supporting facilities and technology. The regulation should also consider non-government owned hospitals, which exemplify most small hospitals. With their limited capacity and the pressure from compulsory accreditation and single-payer insurance policy, a merger could be the only realistic option for small hospitals to cope with when unable to meet the required standard. In addition to overcoming structural barriers, the accreditation process and policies should also be improved. By shifting the emphasis away from administrative compliance, a culture of continuous quality improvement should be encouraged, which is a necessity for a better and sustained hospital performance [61, 62]. This policy approach will maintain the long-term benefit by making the achievement of accreditation status a result rather than a primary aim in and of itself [3].

Based on the current regulation, public reporting of hospital performance indicators is mandatory for all hospitals and is required for applying for or renewing the hospital accreditation status. For private hospitals, it is also a pre-requisite for hospital operational relicensing. Even given this rather strong punitive requirement, the compliance still does not meet expectations which reflected in the small number of hospitals with completed performance indicators. One of the mentioned reasons is a lack of reporting ‘meaningfulness’ for the hospital [63]. Compliance should be viewed as more than a merely administrative requirement [64]. Meaningful and regular feedback for hospitals based on the reported indicator is important to support reporting compliance and data quality for continuous evaluation [64]. The limited evidence on the actual impact of accreditation on hospital performance calls for more rigorous research and long-term evaluation while simultaneously providing a window for continuous monitoring and evaluation.

## Conclusion

The findings from this study regarding the factors associated with hospital accreditation status indicate a strong role for government and the development of a mandatory policy. We found that clinical and efficiency performance among accredited hospitals that was no different from those in non-accredited hospitals may result from the limitations inherent in the hospital performance indicators currently employed. A cautious and in-depth investigation is needed to reveal the underlying factors contributing to the successful adoption of hospital accreditation standards that ultimately leads to continuous performance improvement.

## Additional files

Additional file 1: **Table S1**. Distribution of observed and imputed data. (DOCX 14 kb)

Additional file 2: **Table S2**. Result of the analysis with no imputation. (DOCX 19 kb)

## Data Availability

Permission is not required as data are publicly available and can be accessed online, through the official website of Indonesia Commission on Accreditation of Hospitals, and Hospital Information System, Directorate General of Medical Service, Ministry of Health.

## Abbreviations

ALOS: Average length of stay
BOR: Bed occupancy rate
CQI: Continuous quality improvement
GMR: *Gross mortality rate*
HHI: Herfindahl-Hirschman index
ICAH: Indonesia Commission on Accreditation of Hospitals
IOM: Institute of Medicine
JCI: Joint Commission International
LMICs: Low- and middle-income countries
NMR: *Net mortality rate*
QMS: Quality management system
TOI: Turn over interval
TQM: Total quality management
WHO: World Health Organization
