# Supplementary material for: Hospitals accreditation status in Indonesia: associated with hospital characteristics, market competition intensity, and hospital performance?
Source: BMC Health Serv Res. 2019 Jun 11;19:372. doi: 10.1186/s12913-019-4187-x (PMC6560753; doi:10.1186/s12913-019-4187-x)
Supplement: Supplementary file 1 — Table S1. Distribution of observed and imputed data. (DOCX 14 kb) [file 12913_2019_4187_MOESM1_ESM.docx]

Additional file 1

Table 1. Distribution of observed and imputed data

| **Variables** | **Scale** | **N (missing) %** | **Observed** | **Imputed** |
| --- | --- | --- | --- | --- |
| **Outcome Variables** |  |  |  |  |
| Accreditation status | Accredited | 0 | 217 | NA |
|  | Not accredited |  | 129 | NA |
| Bed Occupancy Ratio | % | 53,2 | 56,06 | 48,61 |
| Average length of stay | days | 56,4 | 4,59 | 4,16 |
| Turn over interval | days | 59 | 5,33 | 5,32 |
| NMR | % | 77,5 | 18,15 | 16,75 |
| GMR | % | 77,2 | 31,46 | 27,50 |
| **Covariables** |  |  |  |  |
| **Organizational design factors** | |  |  |  |
| Hospital size | <=50 | 0 | 108 | NA |
|  | 51-100 |  | 117 | NA |
|  | 101-200 |  | 67 | NA |
|  | >=200 |  | 54 | NA |
| Ownership | Public | 0 | 69 | NA |
|  | Military managed |  | 30 | NA |
|  | State owned enterprise managed |  | 12 | NA |
|  | Private owned |  | 235 | NA |
| Type | General | 0 | 253 | NA |
|  | Maternal and child |  | 67 | NA |
|  | Other range of specialist |  | 26 | NA |
| Number of specialist physician | person | 7,2 | 19,12 | 18,58 |
| **Market competition index** |  |  |  |  |
| Density | % | 0 | 11 | NA |
| Relative size | ratio | 0 | 1 | NA |
| HHI | % | 0 | 42.09 | NA |
