# Supplementary material for: Hospitals accreditation status in Indonesia: associated with hospital characteristics, market competition intensity, and hospital performance?
Source: BMC Health Serv Res. 2019 Jun 11;19:372. doi: 10.1186/s12913-019-4187-x (PMC6560753; doi:10.1186/s12913-019-4187-x)
Supplement: Supplementary file 2 — Table S2. Result of the analysis with no imputation. (DOCX 19 kb) [file 12913_2019_4187_MOESM2_ESM.docx]

Additional file 2. Result with no imputation

**Table 1. Logistic regression analysis of the hospital accreditation status related to organizational design factors (number of beds, ownership status, specialty type, number of specialist), and market density (*with no imputation)***

| **Explored Factors** | **B** | **SE** | **Wald** | **Df** | **Sig*** | **OR** | **95% CI for OR** | |
| --- | --- | --- | --- | --- | --- | --- | --- | --- |
|  |  |  |  |  |  |  | Lower | Upper |
| **Organizational design** |  |  |  |  |  |  |  |  |
| a. Hospital size |  |  |  |  |  |  |  |  |
| - ≤50 (ref.) |  |  | 6.911 | 3 | 0.075 |  |  |  |
| - 51-100 | 0.798 | 0.365 | 4.787 | 1 | 0.029 | 2.222 | 1.087 | 4.543 |
| - 101-200 | 1.476 | 0.599 | 6.077 | 1 | 0.014 | 4.375 | 1.353 | 14.144 |
| - >200 | 2.015 | 1.029 | 3.837 | 1 | 0.050 | 7.501 | 0.999 | 56.327 |
| b. Ownership status |  |  |  |  |  |  |  |  |
| - Public (ref.) |  |  | 9.545 | 3 | 0.023 |  |  |  |
| - Military | 2.218 | 0.802 | 7.648 | 1 | 0.006 | 9.188 | 1.908 | 44.246 |
| - State owned enterprise | 2.082 | 1.169 | 3.172 | 1 | 0.075 | 8.023 | 0.811 | 79.339 |
| - Private | 0.606 | 0.481 | 1.587 | 1 | 0.208 | 1.834 | 0.714 | 4.710 |
| c. Specialty service |  |  |  |  |  |  |  |  |
| - General (ref.) |  |  | 0.863 | 2 | 0.650 |  |  |  |
| - Maternity | -0.431 | 0.471 | 0.835 | 1 | 0.361 | 0.650 | 0.258 | 1.637 |
| - Specialist | -0.252 | 0.525 | 0.231 | 1 | 0.631 | 0.777 | 0.278 | 2.173 |
| 1. Number of specialist physicians | 0.013 | 0.014 | 0.805 | 1 | 0.370 | 1.013 | 0.985 | 1.042 |
| **Market Indicator** |  |  |  |  |  |  |  |  |
| 1. Relative size | 0.681 | 0.479 | 2.025 | 1 | 0.155 | 1.976 | 0.773 | 5.047 |
| Constant | -1.460 | 0.604 | 5.843 | 1 | 0.016 | 0.232 |  |  |

** ref.= reference category*

Hosmer and Lemeshow test p=.29; Nagelkerke R square= .275

**Table 2. The difference of average hospital performance indicators BOR, ALOS, TOI, NMR, GMR from national standards and by accreditation status (*with no imputation*)**

| **Hospital Performance Indicator (n)** | **1**  **National Standard**  **(mid value)** | **2**  **Accredited Hospitals**  **(SE)** | **3**  **Not-accredited Hospitals**  **(SE)** | **p**  **(1 – 2)** | **p**  **(1 – 3)** | **p**  **(2– 3)** |
| --- | --- | --- | --- | --- | --- | --- |
| BOR (162) | 60-85 % (75%) | 56.95 (1.5) | 50.67 (5.1) | <0.001 | <0.001 | 0.140 |
| ALOS (151) | 6-9 days (7.5) | 4.76 (0.6) | 3.66 (0.2) | <0.001 | <0.001 | 0.410 |
| TOI (142) | 1-3 days (2) | 5.33 (0.9) | 5.29 (1.2) | 0.001 | 0.017 | 0.964 |
| NMR (78) | ≤2.5 (2.5) | 18.42 (2.1) | 15.80 (3.9) | <0.001 | 0.004 | 0.583 |
| GMR (79) | ≤4.5 (4.5) | 31.22 (3.1) | 30.5 (7.7) | <0.001 | 0.005 | 0.955 |

*Note: data calculated for the hospital with reported BOR. BOR – bed occupancy ratio; ALOS – average length of stay; TOI – turn over interval; NMR - net mortality rate; GMR – gross mortality rate. For one-sign test we used the mid value of the national standard.*
